# Progress in Surgery

**Published:** 1896-07-04

**Authors:** 


### PROGRESS IN SURGERY.

### GENITO-URINARY SURGERY.

(*Continued from page 210.*)

**Posterior Catheterism as an Aid to Perineal Urethotomy.**—Kukula[10] has collected records of forty-five cases, and operated on five. In cases where it has not been possible to discover the proximal end of the urethra in perineal urethotomy for impermeable stricture, the bladder has been opened and a metal sound passed into this portion of the urethra as a guide to it, and the stricture then divided or excised. In some few cases the same procedure has been carried out in cases of operation for ruptured urethra, when it has not been possible to find the proximal end from the perineum alone. Only four of the cases were fatal, which cannot be considered a high mortality when we remember that either severe injury to the pelvis or cystitis with renal complications were present in these cases.

**Transplantation of Urethra from Sheep.**—A case is recorded by Fenwick, of Christchurch, New Zealand,[11] in which he replaced a large portion of the membranous urethra in this way. The wound healed satisfactorily. It is too early to tell whether stricture will result.

**Rupture of the Urethra.**—Watson records[12] a case of rupture of the penile urethra. The patient received a violent blow from a winch-handle, which drove the penis against the symphysis pubis and crushed it against the bone. Extravasation of urine and sloughing followed, but as late as the fifteenth day the edges of the ruptured part were excised, and the opening sutured with fine silk, free incision being made into the gangrenous tissue, and the bladder drained from a perineal opening. The patient recovered. In another case the rupture occurred in the deep urethra during coitus. It was not complete, and urine was passed with blood after the accident, but rapid extravasation of urine occurred.

**Treatment of Extravasation of Urine by the Continuous Bath.**—Two cases are recorded by Chauncy Puzey,[13] of Liverpool. In both the extravasation and subsequent gangrene of the skin and cellular tissue was so extensive that Mr. Puzey does not think they could have recovered by free incision and antiseptic dressing alone. The first patient was kept in the bath for a week, the second for three weeks. Boracic lotion and sanitas solution were used in the baths. Mr. Puzey does not recommend the treatment—which, he says, is a serious tax upon the surgeons and nurses—in all cases of extravasation of urine, but only in those of exceptional severity.

**Axial Rotation of the Testis.**—Cases of this rare condition have been referred to in previous "Surgical Progress" papers. A case recorded by Sheen, of Cardiff,[14] was not diagnosed at the time it was operated on, but was thought to be an example of hæmorrhagic orchitis. The cord became twisted by the youth jumping over a bridge. No ill-effects were observed at the time, but severe pain set in several hours after, together with tender swelling of the testis to the size of a small orange. An aseptic incision was made into the organ to relieve tension, and the appearance it presented was that of blood clot; the stitches in the scrotal wound gave way a few days later, and a hernia of dead testicular tissue presented and was cut away, and later on the whole organ removed. An examination then showed that the epidedymis was infiltrated with blood from twisting of the cord. In many of these cases where the testis has been undescended strangulated hernia has been suspected. Edmund Owen believes that it usually occurs in cases of undescended testicle. The cord has occasionally been untwisted.

**The Pressure of Bicycle Saddles on the Perineum.**—Attention is called[15] to the harm resulting from such pressure by Townsend. Prostatic and urethral irritability has been produced, and the author fears the occurrence of stricture. A paper on the same subject by Roper, of Exeter, calls attention to the dangers of the triangular-shaped saddle.[16] He has himself experienced from its use frequency of micturition, a cold and shrunken condition of the penis and scrotum, followed by considerable aching.

**Syphilis.**—Dr. Van Niessen, of Wiesbaden,[17] claims to have discovered the bacillus. He finds the organism in the blood, in the form of a diplo-bacillus, taking the form of the letter V, but capable of developing mycelial threads and spores. His inoculation experiments seem hardly to justify his conclusions. A case of phagedæmic true chancre treated by injections of antisyphilitic serum, in this country, is reported by Barling, of Leeds, who considered that the serum had a very good effect. Attention is called to the remedial action of erysipelas in syphilis.[18] Rudolf, of Magdeburg, reports a case of tertiary syphilitic sores on face rapidly healed during an attack of erysipelas, and in another enlarged glands and symptoms of cerebral syphilis disappeared when the patient was attacked by this disease, although mercury and iodide of potassium had failed. Another case in which a complete group of secondary symptoms quickly disappeared during an attack of erysipelas is referred to. Dr. George Ogilvie[19] has collected twenty cases which he considers to be exceptions to Colles' law, that a mother is rendered immune by the birth of a syphilitic child. Dr. Savill[20] draws attention to the existing analogy in the case of small-pox. In the epidemic at Warrington the occurrence of small-pox in pregnant women modified the susceptibility of their offspring to the disease and als) to vaccination, which was the reverse of Colles' law. Jonathan Hutchinson[20] has never seen an exception, though he admits they do very rarely occur, and Drs. Lees and Calcott Fox have not seen an exception,[20] though the former has known the mother infected by a child who had acquired syphilis, chancres forming on the mother's breast.

[10] Klinische Leit und Str-itfragen, Bd. ix., Hft. 5·6.; Epitomy B.M.J., Jan 25, 1896. [11] Lancet, Feb. 8, 1896. [12] International Clinics, vol. iv. [13] Lancet, Feb. 8, 1896, p. 355. [14] Ibid, April 11, 1896, p. 991. [15] Med. Record, Feb. 22, 1696, [16] Lancet, May 16, 1896, p. 1,341. [17] Der Syphilis-Bacillus, 1896; Lancet Jan. 4, 1896, p. 49. [18] New York Med. Journal, Feb. 22, 1896. [19] Brit. Med. J., Feb. 1, 1896, 272. [20] Ibid.

